# Molecular Cloning and Expression of *CYP9A61*: A Chlorpyrifos-Ethyl and Lambda-Cyhalothrin-Inducible Cytochrome P450 cDNA from *Cydia pomonella*

**DOI:** 10.3390/ijms141224211

**Published:** 2013-12-13

**Authors:** Xueqing Yang, Xianchun Li, Yalin Zhang

**Affiliations:** 1 Key Laboratory of Plant Protection Resources & Pest Management of Ministry of Education, Northwest A&F University, Yangling 712100, Shaanxi, China; E-Mail: sling233@hotmail.com; 2 Department of Entomology, University of Arizona, Tucson, AZ 85721, USA; E-Mail: lxc@email.arizona.edu

**Keywords:** *Cydia pomonella*, cytochrome P450, induction, response, insecticides

## Abstract

Cytochrome P450 monooxygenases (CYPs or P450s) play paramount roles in detoxification of insecticides in a number of insect pests. However, little is known about the roles of P450s and their responses to insecticide exposure in the codling moth *Cydia pomonella* (L.), an economically important fruit pest. Here we report the characterization and expression analysis of the first P450 gene, designated as *CYP9A61*, from this pest. The full-length cDNA sequence of *CYP9A61* is 2071 bp long and its open reading frame (ORF) encodes 538 amino acids. Sequence analysis shows that CYP9A61 shares 51%–60% identity with other known CYP9s and contains the highly conserved substrate recognition site SRS1, SRS4 and SRS5. Quantitative real-time PCR showed that *CYP9A61* were 67-fold higher in the fifth instar larvae than in the first instar, and more abundant in the silk gland and fat body than other tissues. Exposure of the 3rd instar larvae to 12.5 mg L^−1^ of chlorpyrifos-ethyl for 60 h and 0.19 mg L^−1^ of lambda-cyhalothrin for 36 h resulted in 2.20-and 3.47-fold induction of *CYP9A61*, respectively. Exposure of the 3rd instar larvae to these two insecticides also significantly enhanced the total P450 activity. The results suggested that *CYP9A61* is an insecticide-detoxifying P450.

## 1. Introduction

The codling moth *Cydia pomonella* (L.) (Lepidoptera: Tortricidae) is one of the most serious pests of pome fruits and walnuts in many countries where these crops are cultivated [1–6]. The fruit damages by *C. pomonella* include boring tunnels to the center of fruit to feed on seeds, contaminating fruits with their frass, and causing fruit abscission. Effective management of the codling moth mainly depends on chemical insecticides [2,5]. A major problem associated with chemical control of the codling moth is its development of resistance to insecticide groups organophosphates (OPs) [7–10] and synthetic pyrethroids (SPs) [11–13] in many countries. Chlorpyrifos-ethyl (OPs) and lambda-cyhalothrin (SPs) are the most commonly used insecticides for codling moth control worldwide and development of resistance to these two insecticides has been reported in the field populations of this species [3,4,10]. In China, however, this introduced pest has not been directly targeted by insecticides and resistance to insecticides has not been reported in most of the affected areas in China.

Cytochrome P450 monooxygenases (CYPs or P450s) are a superfamily of enzymes responsible for detoxification of natural and synthetic xenobiotics including insecticides and plant toxins [14–19]. In the P450 superfamily, CYP6 and CYP9 family members are found in various insect species and play an important role in the detoxification of insecticides and plant toxins in insects. Exposure of insects to plant toxins and/or insecticides often increases the expression of a subset of P450 genes and the total P450 enzyme activity [15,16,20]. Involvements of CYP9 genes in insecticide detoxification and their inductions in response to xenobiotics have been documented in a number of insect pests including *Helicoverpa armigera* (Hübner) [19], *Aphis mellifera* (L.) [17], *Heliothis virescens* (Fabricius) [21], *Aedes aegypti* (L.) [22], *Cimex lectularius* (L.) [23], and *Culex quinquefasciatus* (Say) [24].

In *C. pomonella*, extensive studies have suggested that P450s may play a key role in detoxification of OP and SP due to the observed significantly higher total P450 enzyme activity in the resistant *C. pomonella* strains than in the susceptible laboratory strains [3–5,9,10]. Nevertheless, no single P450 gene has been isolated from this pest, not to mention the precise function of a single P450 gene in insecticide metabolism and resistance. To explore the potential roles of P450 genes in detoxification of insecticides in the codling moth, we cloned the full-length cDNA sequence of the first P450 gene, designed as *CYP9A61*, from this species. The spatiotemporal and insecticide-induced expression profiles of *CYP9A61* revealed by real-time quantitative PCR (RT-qPCR) suggest that it is a xenobiotic-metabolizing P450 gene.

## 2. Results

### 2.1. Identification and Characterization of a Novel Cytochrome P450 Gene (CYP9A61)

**Figure 1.**
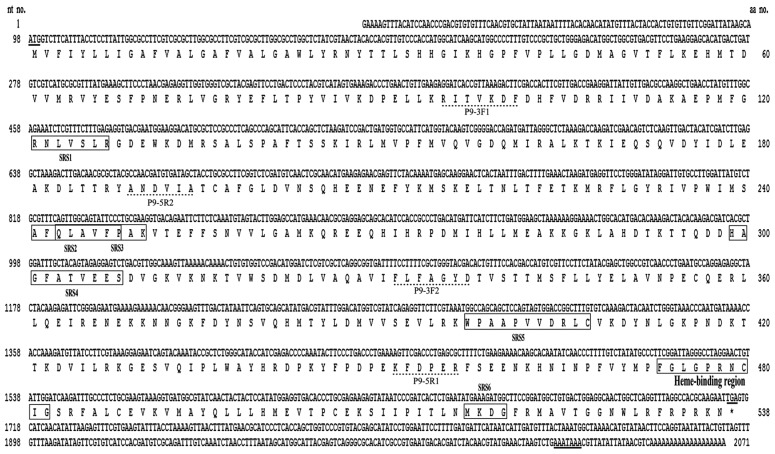
The full-length cDNA sequence of *Cydia pomonella* CYP9A61 and deduced amino acid sequence. The start codon, stop codon, and polyadenylation signal sequences are underlined. The heme-binding domain and six substrate recognition sites (SRSs) are boxed. Dotted lines indicate the domains on which degenerate primers were designed.

A 3′ cDNA fragment of 992 bp and a 5′ cDNA fragment of 688 bp were amplified by 3′ and 5′ RACE using the degenerate primers designed based on the conserved regions of the *CYP9* genes (Table 1). A full-length cDNA of 2071 bp (GeneBank database accession number: KC832920) was then obtained by RT-PCR using the gene specific primers P9F and P9R (Table 1) designed based on the cDNA sequences of the 3′ and 5′ RACE fragments. The full-length cDNA sequence contains a 97 bp 5′-untranslated region (5′-UTR), an open reading frame (ORF) of 1638 bp, and a 357 bp 3′-untranslated region (3′-UTR) (Figure 1). The ORF encodes a protein of 538 amino acids (Figure 1), with a predicted molecular mass of 62.04 kDa and with a theoretical *pI* point of 7.66. The translated protein contains the signature motif of P450s, shares the highest amino acid sequence identity with the members of the CYP9A subfamily, and thus was designated as *CYP9A61* by the Cytochrome P450 Nomenclature Committee.

Amino acid sequence alignment indicates that CYP9A61 shares 58%–60% identity with the CYP9A members from *Bombyx mandarina* (Moore) (CYP9A20, 60%, ACJ05915.1; CYP9A19, 59%, ABQ08710.1; CYP9A21, 58%, ACJ04711.1). It also shares a high degree of amino acids identity with the CYP9A members from *B. mori* (CYP9A20, 59%, NP_001077079.1; CYP9A19, 58%, ABQ08709.1; CYP9A21, 57%, NP_001103394.1), *H. armigera* (CYP9A14, 59%, ABY47596.1; CYP9A12, 58%, ACB30273.2; CYP9A17, 56%, AAY21809.1; CYP9A18, 58%, ABB69055.1), and *Zygaena filipendulae* (L.) (CYP9A36, 57%, ACZ97417.2; CYP9A37, 54%, ACZ97407.2). Moreover, CYP9A61 shares more than 50% identity with CYP9A38 from *Cnaphalocrocis medinalis* (Guenée) (57%, CAZ65619.1), CYP9A39 from *Spodoptera litura* (Fabricius) (57%, ACV88722.1), CYP9A9 from *Spodoptera exigua* (Hübner) (57%, ACI143222.1), CYP9A1 from *H. virescens* (55%, AAD25104.1), and CYP9A5 from *M. sexta* (51%, AAD51038.1).

**Figure 2.**
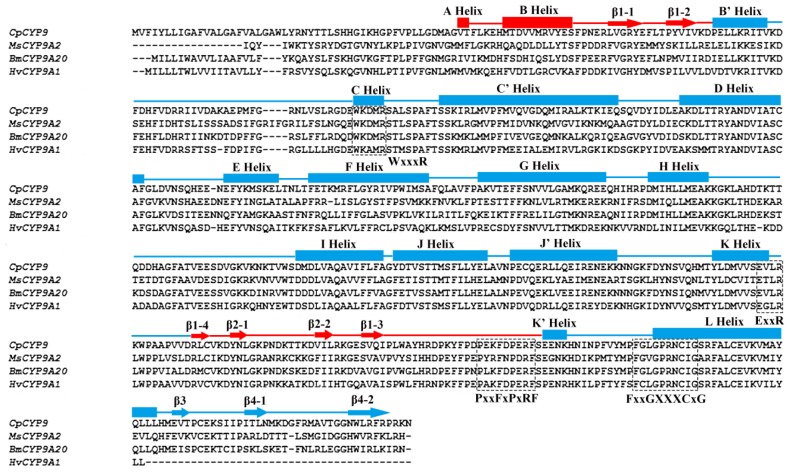
Structure-based sequence alignments of *Cydia pomonella* CYP9A61, *Manduca sexta* CYP9A5, *Heliothis virescens* CYP9A1 and *Bombyx mori* CYP9A20 by ClustalW2. The predicted α-helices and β-sheets of CYP9A61 are marked on the top of sequences using boxes and arrows respectively. The β-domain is red, and α-domain is blue. The conserved regions of P450 enzymes including FxxGxxxCxG, PxxFxPxRF, ExxR, WxxxR are boxed using dashed lines.

The protein contains 16 α-helices, 9 β-sheets and the loops are linked (Figure 2). The second structure of this enzyme is composed of a β-domain (denoted in red: A helix, B helix, β1, β2), and an α-domain (denoted in blue). Motif prediction by scanning the Prosite database shows the CYP9A61 contains a number of typical characteristic features of cytochrome P450 (Figure 2). For instance cytochrome P450 heme-binding domain FxxGxxxCxG (residues 473–482, in which C is the heme iron ligand) is at the *C*-terminal that is conserved in most of insect P450 enzymes. The typical aromatic motif PxxFxPxRF (meander) on the K-K′ loop is found at the position of 449–459 aa in this protein. The ERR-triad formed by the ExxR (residues 392–395) motif and PxRF (residues 454–457) motif may form a set of salt-bridge interactions to assure the cysteine pocket is at the right position to make sure the heme binds to protein [25,26]. The conserved WxxxR (131–135) region located near the *N*-terminal in the C Helix may be involved in eliminating the charge of propionate side chains of the heme [27]. Six substrate recognition sites (SRSs) were also determined (Figure 1) based on the CYP9A61 secondary structure elements [26]. SRS1 is located in the loop B′-C helixes, SRS2 and SRS3 are situated in the helices F and G connecting the loop. In general, SRS4 lies in the center portion of helix I; however, it is located in the loop H-I helixes in this protein. SRS5 is found at the *N*-terminal of β strand 1–4, and β strands 4-1 and 4-2 protrude into SRS6. The CYP9A61 was predicted to have no signal peptide in the protein *N*-terminal but contains a transmembrane domain (residues 2–24) at the *N*-terminus (Figure 1).

### 2.2. Phylogenetic Analysis

**Figure 3.**
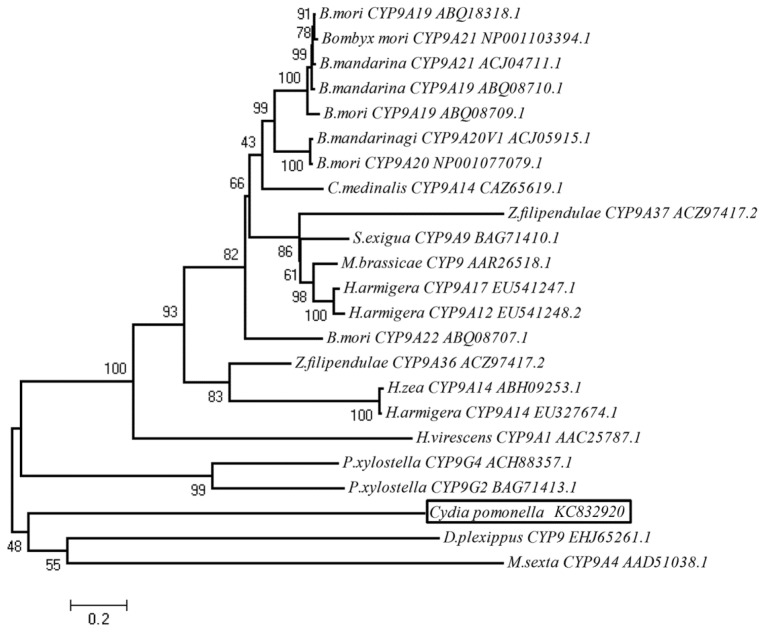
Phylogenetic relationship of *C. pomonella* CYP9A61 with 22 CYP9s from Lepidoptera. CYP9A61 (GenBank accession number: KC832920) is boxed. This un-rooted phylogenetic tree was constructed using the neighbor-joining method. Nodes indicate bootstrap calculated with 1000 replications support.

To examine the relationship of CYP9A61 with members of the CYP9 family from Lepidoptera deposited in the GenBank database, a phylogenetic tree was constructed using the neighbor-joining method (Figure 3). The analysis shows the CYP9A61 is located on the same branch with *Danaus plexippus* (L.) CYP9 and *M. sexta* CYP9A5 with 48% bootstrap support, indicating CYP9A61 shared closest relationship with *D. plexippus* CYP9 and *M. sexta* CYP9A5. Although the Bombycidae’s CYP9s share the highest amino acid identity with CYP9A61 (57%–60%), they are located in another branch with the CYP9s from Noctuidae, Pyralidae and Zygaenidae. In this tree, the transcripts of some CYP9 members were observed to be induced by insecticides. For instance, deltamethrin increased the expression levels of *CYP9A17* and *CYP9A12* from *H. armigera* [26]. The mRNA level of *CYP9A1* from *H. virescens* was increased through exposure to thiodicarb [21].

### 2.3. mRNA Expression of CYP9A in Response to Chlorpyrifos-Ethyl and Lambda-Cyhalothrin Exposure

**Figure 4.**
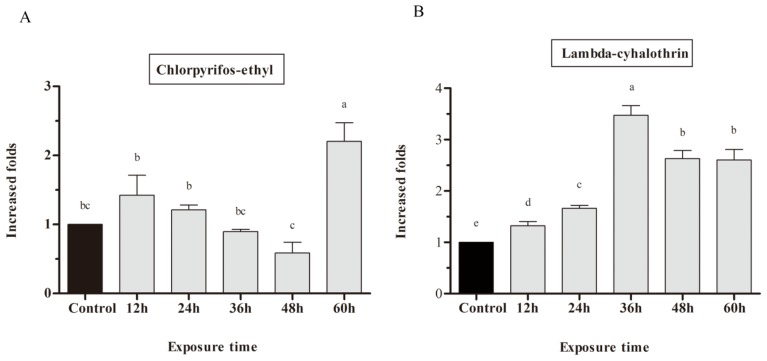
Induction of *C. pomonella CYP9A61*. RT-qPCR analysis relative expression of CYP9A61 treated with 12.5 ng chlorpyrifos-ethyl (**A**) and 0.19 ng lambda-cyhalothrin (**B**). Expression level was first normalized to the reference gene beta*-actin*. The normalized value of each sample was then divided by a specified control (the value of CYP9A61 in acetone treated control), and the ratio was applied to relative expression analysis. The results are shown as the mean ± SE. The error bars show the ranges of standard errors. Letters on the error bars indicate the significant differences by ANOVA analysis (*p* < 0.05).

In this study, we investigated if chlorpyrifos-ethyl and lambda-cyhalothrin are inducers and if CYP9A61 is involved in detoxification of insecticides. Third instar larvae were treated with chlorpyrifos-ethyl and lambda-cyhalothrin, and their mRNA expression levels were detected by RT-qPCR. After treatment with chlorpyrifos-ethyl on the dorsum of the larvae, no significant difference in *CYP9A61* expression was observed in the first 48 h. The 60 h chlorpyrifos-ethyl exposure led to a 2.2-fold up-regulation of CYP9A61 at a concentration of 12.5 ng when compared with the control (Figure 4A). The *CYP9A61* transcript level was strongly induced after exposure to lambda-cyhalothrin as time progressed from 12 to 36 h (approximately 1.32–3.47 fold), and thereafter dropped to 2.60 fold after 60 h of lambda-cyhalothrin exposure (Figure 4B).

### 2.4. Developmental Expression Profiles

**Figure 5.**
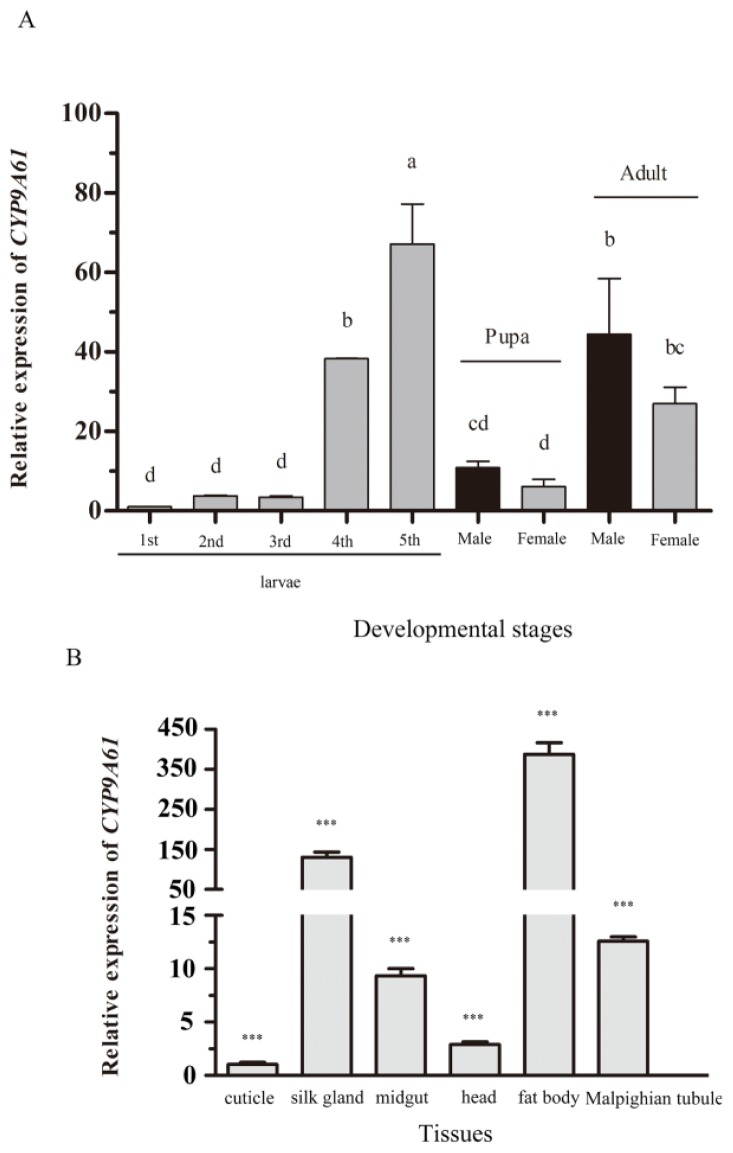
Relative expression level of *C. pomonella CYP9A61* in different developmental stages (**A**) and tissues (**B**). Expression level was normalized using β-actin as the standard. The normalized value was applied to relative expression analysis. The results are shown as the mean ± SE. The error bars show the ranges of standard errors. Letters or asterisks on the error bars indicate the significant differences by ANOVA analysis (*p* < 0.05) or by Student’s *t*-test (*** *p* ≤ 0.001).

To determine the relative expression patterns of *CYP9A61* mRNAs at the various developmental stages, a RT-qPCR was conducted. The *CYP9A61* transcript was detectable in all developmental stages, and was significantly higher in the fifth instar than at other ages (Figure 5A). The results showed that no significant difference in *CYP9A61* expression was observed from first to third instar. There was a rapid increase of *CYP9A61* expression at the fourth instar of *C. pomonella*, and it reached a peak in fifth instar larvae (67-fold of the first instar). The gene expression level dropped at the pupal stage (10.85 and 6.15-fold of the first instar for male and female, respectively) and finally increased in adults (44.44 and 26.94-fold in male and female, respectively). Interestingly, the expression of *CYP9A61* was not different between the male and female codling moth in pupal and adult stages.

### 2.5. Tissue Specific Expression Profiles

The mRNA expression levels of the *CYP9A61* in the head, cuticle, silk gland, midgut, fat body, and Malpighian tubule dissected from third instar larvae were investigated (Figure 5B). The results showed that *CYP9A61* was expressed in all tissues (Figure 5B). Figure 5B shows that the *CYP9A61* has a high level of mRNA expression in silk glands (127.78-fold higher than in the cuticle) and fat body (384.00-fold higher than in the cuticle), while the gene has relative low expression in midgut, head and the Malpighian tubule, at 9.87-, 3.61-, and 12.58-fold of the cuticle (Figure 5B).

### 2.6. Enzyme Activity

**Figure 6.**
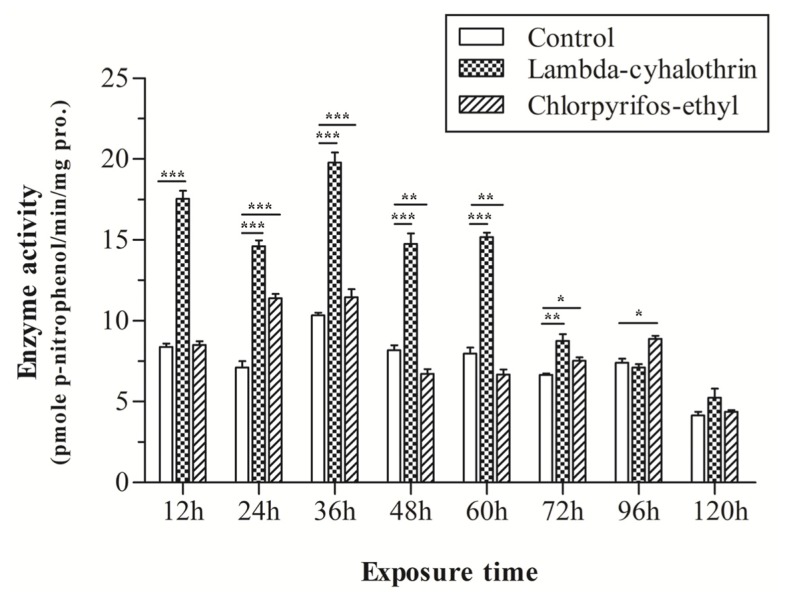
MFO activities of *C. pomonella* treated with 2.5 ng chlorpyrifos-ethyl and 0.19 ng lambda-cyhalothrin using PNOD as substrate. The results are expressed as pmole *p*-nitrophenol min^−1^ mg of protein^−1^. The error bars represent the standard error across three replicates. Asterisks on the error bars indicate the significant differences between chlorpyrifos-ethyl or lambda-cyhalothrin treatment and control by Student’s *t*-test. *p ≤ 0.05; **p ≤ 0.01; ***p ≤ 0.001.

In order to determine whether chlorpyrifos-ethyl and lambda-cyhalothrin could influence the mixed-function oxidase (MFO) activity, the MFO activities using *p-*nitroanisole as substrate are shown in Figure 6. The between-subjects effects (insecticide exposure and exposure time) were analyzed using a factorial ANOVA (Table 2). A significant difference in *p*-nitroanisole demethylase (PNOD) activity was observed after treatment with chlorpyrifos-ethyl (Table 2, Figure 6). The PNOD activity was no different between the treated group and control after 12 h treatment, but significantly increased after 24 and 36 h treatment, and then fell after exposure for 48 h. The PNOD mean activity for 24 h chlorpyrifos-ethyl-exposure is 11.55 pmole min^−1^ mg protein^−1^, and the value is 7.14 pmole min^−1^ mg protein^−1^ for the control, which constitutes a 1.62-fold increase. A weak activity (0.83, and 0.83-fold compared to control) was observed for the experimental group at 48 h and 60 h, with the value of 6.75 and 6.66 pmole min^−1^ mg protein^−1^, respectively. Higher activity was detected for the longer time inductions (72 and 96 h), with 1.14 and 1.20-fold of PNOD activity increase when compared with the control.

For the lambda-cyhalothrin exposed larvae, the main interaction effects for insecticide exposure and exposure time and insecticide exposure and exposure time were significant (Table 2). Significantly higher PNOD activities were exhibited after exposure to lambda-cyhalothrin in the previous 72 h compared with the control, and then declined to the same level as the control (Figure 6). The results indicate that the PNOD activity was 2.09-, 2.05-, 1.98-, 1.81-, 1.90- and 1.31-fold higher than the control after exposure to lambda-cyhalothrin as time progressed from 12 to 96 h, respectively. No difference was observed after 96 h of lambda-cyhalothrin exposure.

## 3. Discussion

**Figure 7.**
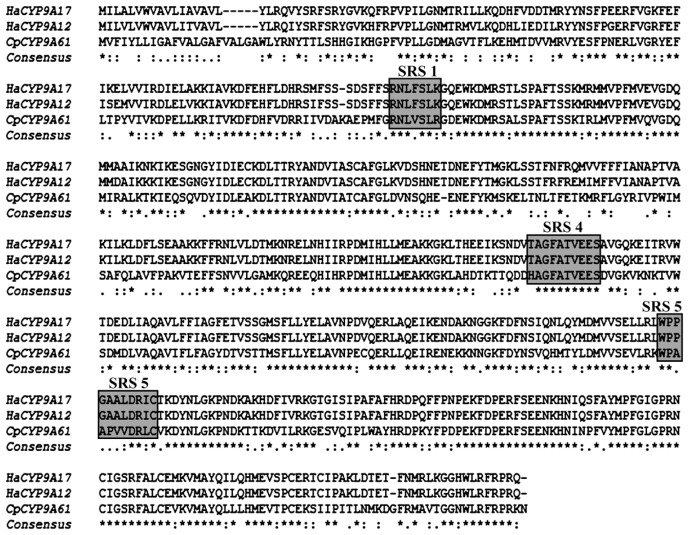
Comparison of *C. pomonella* CYP9A61 and CYP9A12 (EU541248.2) and CYP9A17 (EU541247.1) from *Helicoverpa armigera*. SRS1, SRS4 and SRS5 are boxed. Asterisks (*) indicate identical residues among three sequences; colons (:) indicate residues with conserved substitutions; dots (.) represent residues with weakly conserved residues in the three sequence alignment.

The great interest in insect P450s is due to their role in detoxification of natural and synthetic endogenous and exogenous compounds including insecticides and plant toxins [14–19]. *CYP9A61* cloned from *C. pomonella* larvae in the present study represents the first P450 gene isolated from this species. Sequence alignment demonstrates that CYP9A61 shares 57% and 56% homology with *H. armigera CYP9A12* and *CYP9A17*, respectively. In addition, the SRS1, SRS4 and SRS5 are highly conserved with the CYP9 members above (Figure 7). In *H. armigera*, *CYP9A12* and *CYP9A17* were potentially involved in xenobiotic metabolism [26]. It is noteworthy that CYP9A61 shares a 55% amino acid identity with *H. virescens* CYP9A1 and 51% with CYP9A5 from *M. sexta*; these two P450s were found to be induced by thiodicarb [21] and clofibrate [28], respectively. Therefore, we infer that the CYP9A61 in codling moth could also be induced by insecticide exposure. Based on the high sequence identity and second structure similarity, we infer that the CYP9A61 have the same function in metabolism of endogenous and exogenous compounds including insecticides, fatty acids and plant toxins.

We investigated the *CYP9A61* transcription levels in different development stages. Interestingly, the results show the highest value in fifth instar larvae and levels then dropped in the pupa and adult which is congruent with previous studies in a NADH-cytochrome b5 reductase from *H. armigera* [29], suggesting that the *CYP9A61* expression profile during the life cycle is consistent with that of co-regulated functional elements of the microsomal drug oxidation system. Furthermore, the expression pattern is also in line with that in the Diptera insect, *C. quinquefasciatus* [24]. No significant difference in expression of *CYP9A61* was observed among males and females in both pupa and adult stages, suggesting that *CYP9A61* expression may not be related to gender in these stages. However, *CYP9A12* from *H. armigera* was found to have a higher expression level in both the pupal and adult male than in the female [26]. Furthermore, CYP transcription profiles of some *CYP6s* from Dipteran species, such as *Drosophila melanogaster* (Meigen) [30] and *CYP6Z1* from *Anopheles gambiae* [31] where there is a significant difference of gene expression among males and females, may be related to the gender. Unfortunately, sexing the larvae is unfeasible and whether CYP9A61 is related to gender in our larvae was uninvestigated. The gene expression patterns of P450s in different developmental stages were related to the biological and physiological function of P450s. Our results reveal that *CYP9A61* expression levels in the whole body of the codling moth are higher in the active feeding stages (fourth and fifth instar larvae) than non-feeding stages (pupa and adult). This is somewhat consistent with CYP4 genes from fat body and midgut of *M. sexta* [32]. In that case, the expression levels of CYP4 genes were higher in active feeding, midwandering, prepupal, and pupal stages. This expression pattern suggests that *CYP9A61* is potentially responsible for detoxification of xenobiotic compounds including insecticides or plant toxins suffered during feeding.

To better understand the function of CYP9A61, the distribution of *CYP9A61* in the tissues of the codling moth was determined. It has been reported that tissue-specific expression of detoxification related genes might illustrate some of the functions of their biological and physiological roles [31]. We found similar developmental expression profiles of *CYP9A61* with NADH-cytochrome b5 reductase from *H. armigera*, but the tissue-specific expression pattern of *CYP9A61* is inconsistent with the *H. armigera* NADH-cytochromeytochroreductase [29]. To our knowledge, the fat body in insects is a key tissue associated with energy metabolism, detoxification of xenobiotics and intermediaries [33,34]. In our study, the high expression level observed in fat bodies further indicates that *CYP9A61* may be associated with detoxification of xenobiotic compounds. Silk glands, an organ which produce threads of silky material, where *CYP9A61* is abundantly expressed, indicates that *CYP9A61* has a potential role in protection of codling moth from mechanical injuries. However, to determine the accurate function of CYP9A61 in detoxification of xenobiotic compounds, the *CYP9A61* expression level of specific-tissues of codling moth exposure to insecticides should be further investigated.

In insects, substantial experimental effort is being focused on the P450s in response to plant allelochemicals and other secondary metabolites to elaborate plant-insect co-evolution interactions [18,35]. The specific P450 genes acting in response to insecticide exposure are less known [36], especially in the codling moth. A previous study demonstrated that the MFO activities for all stages of whole larvae in a pyrethroid resistant codling moth population were significantly increased compared to a susceptible strain [37]. However, inducibility is one of the characteristics of P450s; many of them can be induced by xenobiotic compounds [15,38]. In a thiodicarb selected *H. virescens* strain, the expression of *CYP9A1* from whole body was 29- and 13-fold higher than in two susceptible strains [21]. Elevated *CYP9* mRNA transcripts for all developmental stages were observed in pesticide-exposed *C. lectularius* collected under repeated insecticide treatments in an apartment in Columbus, OH, USA [23]. In *A. mellifera*, *CYP9Q3* in the midgut of 3-d-old worker bee was slightly induced (1.5-fold) by tau-fluvalinate [17]. A similar phenomenon has been reported for *CYP9M8* (2.5-fold) and *CYP9M9* (2-fold) in permethrin-exposed fourth instar *A. aegypti* larvae [22]. Furthermore, in *H. armigera*, deltamethrin induced *CYP9A12* and *CYP9A17* expression in the midgut whereas it repressed both *CYP9A12* and *CYP9A17* transcripts in fat body [26]. Based on this evidence, we hypothesize that lambda-cyhalothrin and chlorpyrifos-ethyl can also induce *CYP9A61* in the codling moth.

In this study, the response of *CYP9A61* to chlorpyrifos-ethyl and lambda-cyhalothrin exposure was investigated. The *CYP9A61* transcript is significantly induced by lambda-cyhalothrin (Figure 4B) using the dosage selected, and the same tendency is shown in the enzyme activity assay (Figure 6). Many CYP genes belonging to the CYP9 family were induced by synthetic pyrethroid insecticides. In *D. melanogaster*, xenobiotic inducibility of the P450 genes is associated with insecticide resistance [30]. In pyrethroid-selected *A. aegypti* strains, more than 13 CYP9s were observed up-regulated [39]. González *et al*. observed a high level of CYP in fenitrothion- and pyrethroid-resistant populations [40]. Yang *et al*. demonstrated that *CYP9A12* and *CYP9A14* from *H. armigera* are up-regulated in pyrethroid resistant strains [41]. These suggest that the CYP9 family is induced by synthetic pyrethroids. In many cases, overexpression of P450s often leads to increased metabolism of pesticides, and increased enzyme activity often enhances detoxification [42]. In this experiment, both the molecular and biochemical data indicate that *CYP9A61* is strongly induced by exposure to lambda-cyhalothrin, suggesting that CYP9A61 is a critical protein potentially associated with lambda-cyhalothrin metabolism. Further studies should be carried out to verify the function of CYP9A61 using RNAi to knock the transcript level down or metabolize lambda-cyhalothrin *in vitro*.

In the present study, variations between *CYP9A61* expression levels and PNOD activity post exposure were not precisely consistent with each other, especially for chlorpyrifos-ethyl. For chlorpyrifos-ethyl, the *CYP9A61* transcript is 2.2-fold increased by up to 60 h exposure (Figure 4A), whereas PNOD activity was remarkably increased after 24 h of insecticide induction and weakly increased at 36 h, with subsequent activity repressed after 48 h treatment (Figure 6). Another peak appeared at 72 h. The observed differences between gene expression and enzymatic activity demonstrate that the inducibility of *CYP9A61* transcripts is not precisely consistent with enzyme activity. A similar finding has been documented for *CYP9Q1*, *CYP9Q2* and *CYP9Q3* in *A. mellifera* [17]. We suspect that the reason for no direct correlation between PNOD enzymatic activity and single CYP gene expression could be that cytochrome P450-mediated *O*-demethylation activity is under polygenic control. It’s also worth pointing out that each species contains numerous P450s [43]. Although the number of P450s expressed in the codling moth is not certain, there are numerous P450s expressed in third-instar larvae. In the present work, only the expression level of *CYP9A61* was investigated. In order to clarify the MFO activity variations correlated with CYP6A61 transcript variations, more P450 genes should be further investigated. This phenomenon could also be explained by Serikawa’s viewpoint that there is not always a correlation between transcript level changes and translation changes [44]. Gene expression is a complex process that is regulated at several levels and regulation can occur in the post-transcriptional step [45]. However, the increased CYP levels in mRNA transcripts and enzymatic activity may provide evidence in support of *CYP9A61* potential involvement in the metabolism of chlorpyrifos-ethyl, and this may be a common detoxification mechanism for CYP9s induction in response to insecticides. The low increased ratios observed in both mRNA expression level and enzyme activity are due to the low induction dose selected. The differences of *CYP9A61* response to these two insecticides may be explained by the differences in insecticides and by the treatment dosages used.

## 4. Experimental Section

### 4.1. Insects

The apples with tunnels and frass produced in the skin after consumption by *C. pomonella* larvae were collected from abandoned apple orchards in Wuwei City (Gansu Province, China), a non-managed codling moth affected area. Low instar *C. pomonella* larvae were reared on the original collected whole apples. The higher instar larvae were removed from the apples and reared on flesh of the apple debris in the laboratory at 16:8 h light:dark photoperiod, 25 ± 1 °C and 60% ± 5% relative humidity [5] without exposure to any insecticides and chemicals. The larvae were reared until the adult stage for mating to obtain the next generation of neonates. This codling moth strain reared in the laboratory for 6 generations was used in this study.

### 4.2. Insecticides and *C. pomonella* Treatment

Chlorpyrifos-ethyl and lambda-cyhalothrin with purities of >99% were obtained from Aladdin Reagent (Shanghai, China). These insecticides were dissolved in acetone. Rodríguez *et al*. demonstrated that the *LC*_50_ of chlorpyrifos-ethyl and lambda-cyhalothrin on neonate codling moth were 157.65 mg/L and 0.35 mg/L [4], respectively, when the insecticides are applied on the surface of a 4 cm^2^ semi-artificial diet piece. In the present study, the topical application method [10] was used. The induction doses using topical application method for these two insecticides for codling moth have not been defined previously. A non-lethal dose, 12.5 mg/L for chlorpyrifos-ethyl and 0.19 mg/L for lambda-cyhalothrin was used as the inducing dose for the gene induced-expression and enzyme assay. Larvae were starved for 12 h, and a 1 μL of chlorpyrifos-ethyl solution (12.5 ng) and lambda-cyhalothrin solution (0.19 ng) was applied on the dorsum of each larva according to this topical application method [10] using an Eppendorf pipettor (Hamburg, Germany). Acetone was used for the control. After treatment, larvae were supplemented with the flesh of apple pieces as food and kept under the conditions described above. Each experiment was performed in triplicate, and for each treatment in each time point, five third-instar larvae were used. Insecticide and acetone treated larvae were collected at 12, 24, 36, 48, 60 h for investigation of the mRNA expression level and enzymatic study. First to fifth instar larvae, male and female pupae and adults were sampled from untreated controls. Each assay was carried out on 3 groups of 5 individuals. The larvae were not sexed due to this being unfeasible at that life stage. Head, cuticle, silk gland, midgut, fat body, and Malpighian tubules were collected from 3 groups of 30 third instar larvae. Each group was flash frozen in liquid nitrogen and kept at −80 °C for RNA extraction or enzyme preparation later.

### 4.3. RNA Extraction and cDNA Synthesis for Molecular Cloning

The total RNA of *C. pomonella* was extracted from five third instar larvae using the RNAiso Plus Kit based on the manufacturer’s instructions (Takara, Dalian, China). The quality and concentration of extracted RNA was examined by agarose gel electrophoresis and spectrophotometer analysis (Infinite M200 PRO, Tecan Group Ltd., Männedorf, Switzerland). The RNA samples were then digested with DNase I (MBI) to remove the genomic DNA and for cDNA synthesis. For 3′-rapid amplification of cDNA ends (3′RACE), first strand cDNA was synthesized from 1 μg of total RNA using the SMART™ RACE cDNA Amplification Kit and MLV Reverse Transcriptase (Clontech, Dalian, China) as described by the manufacturer. For 5′-rapid amplification of cDNA ends (5′RACE), single-stranded cDNA was synthesized from 1 μg of total RNA by 5′-Full RACE Kit (Takara, Dalian, China) according to the procedure recommended. The product was stored at −20 °C for future use.

### 4.4. Molecular Cloning of CYP9A61 cDNA by 3′ and 5′RACE

Degenerate primers used for 3′ and 5′RACE amplification were designed (Table 1) based on the conserved amino acid sequences of the insects’ CYP9 family deposited in the NCBI database using the CODEHOP web tool [46]. 3′RACE PCR was conducted by nested PCR using P9-3F1 and 10× Universal Primer Mix (UPM, Clontech, Dalian, China) as the primer pair for the first round PCR reaction and P9-3F2 combined with 10× UPM for the second round amplification. For 5′RACE, PCR was carried out by nested PCR using P9-5R1 and Outer Primer as the primer pair for first round PCR amplification and followed by the second round amplification using P9-5R2 combined with Inner Primer (Table 1). The Outer and Inner Primers were supplied by 5′-Full RACE Kit. The PCR was conducted in a C1000 Thermal Cycler (BioRad, Hercules, CA, USA). For both 3′ and 5′RACE, the PCR conditions for the first round PCR reaction were: 94 °C for 3 min, followed by 35 cycles of 30 s at 94 °C, 30 s at 55 °C and 2 min at 72 °C, and then a final extension at 72 °C for 7 min. For the second round PCR, PCR reaction conditions were the same except the extension time was shortened to 1 min. The PCR products were separated on a 1% agarose gel electrophoresis and the bands with approximately 1000 bp (for 3′RACE) and 700 bp (for 5′RACE) were gel purified using the Biospin Gel Extraction Kit (Bioer Technology Co., Ltd., Hangzhou, China) as described by the manufacturer. The purified fragments were ligated into the pMD-19 T vector (Takara, Dalian, China) and ten positive clones were sequenced (Shanghai Sunny Biotech Co., Ltd., Shanghai, China). Only one 3′ and 5′ cDNA end were obtained using those primers. The sequenced clones did not show any SNPs. Based on the obtained sequences of the 3′ and 5′RACE, the putative full-length of *CYP9A61* gene was amplified with specific primers P9F and P9R (Table 1). The PCR was conducted using high-fidelity Ex *Taq* polymerase (Takara, Dalian, China) to eliminate any potential error occurring by *Taq* DNA polymerase. The full-length cDNA sequence was subjected to the Open Reading Frame Finder (ORF Finder, http://www.ncbi.nlm.nih.gov/gorf/gorf.html) for open reading frame (ORF) prediction. The ORF of CYP9A61 cDNAs was amplified by using primer pairs P9ORF-F and P9ORF-R (Table 1). The PCR was performed at 94 °C for 3 min, followed by 35 cycles of 94 °C for 30 s, 55 °C for 30 s and 72 °C for 2 min, with a final extension at 72 °C for 7 min. The PCR product was TA cloned and sequenced as described above.

### 4.5. Sequence Analysis

The CYP9A61 amino acid sequence was deduced from ORF cDNA sequence using the ExPASy Proteomics web tool [47]. Using this web tool, the molecular weight (Mw) and theoretical isoelectric point (*pI*) was also predicted. The deduced amino acid sequence of CYP9A61 was aligned with *Manduca sexta* CYP9A5 (GeneBank number: AAD51038.1), *H. virescens* CYP9A1 (GeneBank number: AAC25787.1) and *Bombyx mori* (L.) CYP9A20 (GeneBank number: NP_001077079.1) which have been reported as possibly induced by insecticide exposure [21] using the ClustalW2 software [48]. The secondary structure prediction was performed with PSIPRED [49]. Motifs were predicted based on scanning the Prosite database [50] The presence of signal peptide was predicted by SignalP 3.0 [51] and transmembrane was determined using the Simple Modular Architecture Research Tool [52]. An un-rooted tree was constructed by MEGA 4 [53] under the Poisson-correction model using the neighbor-joining method with 1000 replications support.

### 4.6. Real Time Quantitative PCR (qPCR)

QPCR was carried out to study tissue distribution, stage specific and insecticide-induced expression of *CYP9A61*. The total RNA was extracted from collected tissues, the whole body of various developmental stages of *C. pomonella*, and insecticide and acetone (control) larvae. For developmental expression and insecticide induction expression assays, five individuals were used to isolate total RNA, using three independent extracts. For the tissue specific expression study, each tissue dissected from 30 third instar larvae was regarded as an independent specimen to isolate total RNA, using three independent extracts. The first strand cDNA of each RNA sample for qPCR was synthesized using 1.5 μg of total RNA using PrimeScript™ RT reagent Kit with gDNA Eraser (Takara, Dalian, China) in a 20 μL reaction mixture as described by the manufacturer. The relative expression levels of *CYP9A61* in different tissues, at each developmental stage, and under chlorpyrifos-ethyl and lambda-cyhalothrin exposure were assessed using the reference gene *C. pomonella* beta-actin (*CpActin*, GenBank number: KC832921) as a reference. In our previous study, *CpActin* was showed to be an excellent reference gene due to being expressed constantly in different tissues, developmental stages, and exposure to insecticides [54]. The primers used were QCYP9A61F and QCYP9A61R (Table 1) for target gene amplification, and QActinF and QActinR (Table 1) for endogenous control. The qPCR was performed on a BioRad iCycler iQ5 (BioRad, Hercules, CA, USA) using an UltraSYBR Mixture (CWBIO, Beijing, China) with 20 μL reaction mixture consisting of 10 μL UltraSYBR Mixture, 1.5 μL cDNA, 0.1 μL of each 10 μM forward and reverse primers, and adding distilled water up to 20 μL under the following cycling program: 1 cycle at 95 °C for 10 min, 40 cycles at 95 °C for 15 s, 53 °C for 1 min, and a melting curve program (55 to 95 °C in increments of 0.5 °C every 20 s) was conducted after PCR to confirm if the nonspecific product was produced. The standard curves of both *CYP9A61* and *CpActin* were obtained using a linear gradient dilution (10-fold dilution) of cDNA as template. The amplification efficiencies, slopes, and correlation coefficients (*R*^2^) were 100.7%, −3.306 and 0.994 for CYP9A61 standard curve, and 95%, −3.334 and 0.995 for *CpActin*, respectively. The relative *CYP9A61* mRNA expression was calculated according to the 2^−ΔΔCt^ method [55]. A negative control (NTC) reaction was carried out using RNase-free water instead of cDNA templates. A no transcription control (NRC) was also conducted to eliminate DNA contamination. Each experiment contains three biological replicates, and each sample was technically repeated three times.

### 4.7. Enzyme Activity

Whole bodies from insecticide-treated and acetone-treated third instar larvae were homogenized on ice for three replications of five individuals each in 2 mL ice-cold 0.1 M sodium phosphate buffer (pH 7.8) containing a final concentration of 1 mM phenylmethylsulfonylfluoride (PMSF), 1 mM ethylene diamine tetraacetic acid (EDTA) and 1mM dithiothreitol (DTT). The homogenates were centrifuged at 4 °C for 20 min at 12,000 g, and the supernatants were used as an enzymatic source for the MFO assay [56]. The proteins were quantified by the Bradford method [57] using bovine serum albumin as the standard.

The MFO activities were assessed by measuring PNOD activity on Nunc 96-well transparent microplates (Nunc, Roskilde, Denmark) as described by Yang *et al*. [56] with slight modification. The reaction mixture consisted of 180 μL of 0.1 M sodium phosphate buffer (pH 7.8) containing 2 mM *p*-nitroanisole, 10 μL of enzyme. After 5 min of incubation at 30 °C, the reaction was initiated by adding 10 μL of 9.6 mM reduced form of nicotinamide adenine dinucleotide phosphate (NADPH). A negative control reaction was conducted using heat inactivated enzyme (95 °C for 10 min) to eliminate any non-enzymatic activity. Moreover, a no-NADPH-added control was also performed. Pre-incubated sodium phosphate buffer was used for the blank. After 1 h of reaction at 30 °C, the absorbance was read in an Infinite M200 PRO Microplate Reader (Tecan, Männedorf, Switzerland) at 405 nm. A standard curve was conducted with *p*-nitrophenol, and the result was expressed as pmole *p*-nitrophenol min^−1^ mg of protein^−1^. Each experiment contains three biological replicates and three replicates were conducted for each test and the average was used for statistical analysis.

### 4.8. Statistical Analysis

Statistical analysis was carried out by the SPSS statistics software (version 12, IBM, IBM Inc., Chicago, IL, USA). One-way analysis of variance (ANOVA, *p* = 0.05) was used for statistical analysis to determine the significance of the difference in *CYP9A61* mRNA expression for developmental stages and insecticide induction in the codling moth. The differences in *CYP9A61* mRNA expression for tissue distribution was assessed by Student’s *t*-test (* *p* ≤ 0.05; ** *p* ≤ 0.01; *** *p* ≤ 0.001). Factorial ANOVA and Student’s *t*-test were used to analyze the enzyme activity of *C. pomonella* treated with different insecticides and different durations of exposure to insecticide. The results were plotted using GraphPad Prism 5 (GraphPad Software, San Diego, CA, USA).

## 5. Conclusions

In summary, this is the first sequence of a P450 gene isolated from *C. pomonella*. We can conclude that *CYP9A61* is a xenobiotic-metabolizing P450 gene. This study advances our understanding of *CYP9A61* function, and regulation mechanism, and the *CYP9A61* response to insecticide exposure. Further studies of CYP9A61 detoxification of xenobiotic compounds *in vitro* are required for accurate assessments of the metabolic functional activity toward these chemicals.

## Figures and Tables

**Table 1.** PCR primers used in this study.

Name	Sequence (5′-3′)	Primer used
P9-3F1	GGTCAAGCAGATCACCGTGaarganttyga	3′RACE
P9-3F2	GGCCTTCCTGTTCTTCTTCgsnggntwyga	3′RACE
oligod(T)	AAGCAGTGGTATCAACGCAGAGTAC(T)_18_VN	3′RACE
10 × UPM	Long: CTAATACGACTCACTATAGGGCAAGCAGTGGTATCAACGCAGAGT	3′RACE
	Short: CTAATACGACTCACTATAGGGC	3′RACE
P9-5R1	CCGCTCGGGGTCGaayttnthngg	5′RACE
P9-5R2	GGCGCAGGTGGCGatnacrtcrtt	5′RACE
Outer Primer	TACCGTCGTTCCACTAGTGATTT	5′RACE
Inner Primer	CGCGGATCCTCCACTAGTGATTTCACTATAGG	5′RACE
P9ORF-F	ATGGTCTTCATTTACCTCCT	ORF cloning
P9ORF-R	TCAATTCTTGCGTGGCCTAAAC	ORF cloning
P9F	GAAAAGTTTACATCCAACCCGAC	Full-length cloning
P9R	GACGTTATAATATAACGTTTATTTCAG	Full-length cloning
QCYP9F	AAATACCGCTCTGGGCATAC	qPCR
QCYP9R	GATACGCCATCACCTTTACTT	qPCR
QActinF	CGGCATCCACGAAACCACCT	qPCR
QActinR	TGGAAGGAGCCAGTGCGG	qPCR

**Table 2.** Variance analysis of factors influencing PNOD activity induced by two insecticides on *Cydia pomonella* larvae (between-subjects effects).

Source	*d.f.*^a^	Mean square	*F*	*p*^b^
Chlorpyrifos				
Insecticide exposure	1	10.694	22.775	0.000
Exposure time	7	44.106	99.932	0.000
Insecticide exposure × exposure time	7	9.781	20.831	0.000
Error	80	0.470		
Lambda-cyhalothrin				
Insecticide exposure	1	288.956	745.795	0.000
Exposure time	7	138.226	150.066	0.000
Insecticide exposure × exposure time	7	44.372	47.087	0.000
Error	80	0.921		

a Degree of freedom;

b Distinctness index, *p* < 0.05 indicates that there are significantly different.
